# Consumo de Oxigênio e Aptidão Cardiorrespiratória | Diferença entre Idade Cronológica e Biológica

**DOI:** 10.36660/abc.20200648

**Published:** 2020-09-18

**Authors:** Artur Haddad Herdy, Amberson Vieira Assis

**Affiliations:** 1 Instituto de Cardiologia de Santa Catarina São José SC Brasil Instituto de Cardiologia de Santa Catarina – Cardiologia, São José, SC – Brasil

**Keywords:** Atividade Motora, Exercício, Classe Social, Atletas, Treino Aeróbico, Resistência Física, Estilo de Vida Saudável, Sedentarismo, Isquemia Miocárdica/prevenção e controle

O oxigênio é, sem dúvida, um dos principais elementos para a vida de um organismo aeróbico. Os animais com estruturas mais complexas – dentre eles, os seres humanos – desenvolveram no processo evolutivo o metabolismo oxidativo como principal fonte de produção de energia. Sendo assim, a nossa capacidade de aproveitamento e utilização do oxigênio representa a principal usina de energia e está intimamente associada à nossa vitalidade. A sua aferição deveria ser considerada mais um sinal vital. Na avaliação de atletas ou de pessoas com patologias graves, o consumo de oxigênio (VO_2_) é um importante marcador de desempenho ou sobrevida.^1^ O VO_2_ também define terapias fundamentais em pacientes graves com ICC. Em uma série de 715 pacientes encaminhados para transplante cardíaco, a sobrevida livre de eventos como morte e transplante em 1 ano foi de 87% diante de VO_2_ >14, 77% entre 10,1 e 14 e 65% quando ≤10 mL/kg.min.^2,3^ Por outro lado, valores acima de 20 mL/kg.min são considerados de melhor prognóstico e podem seguir em terapia farmacológica.^4^ Recentemente, na pandemia do coronavírus-19 (Sars-CoV-2), o valor dos níveis arteriais de oxigênio se mostrou importantíssima ferramenta na avaliação da gravidade desses pacientes. Nos quadros iniciais de lesão pulmonar, os pacientes apresentavam-se relativamente bem, apesar da dessaturação de oxigênio pouco responsiva à oxigenioterapia, em um claro descolamento entre a gravidade da hipoxemia e os sintomas, o que tem sido descrito como “*Happy hypoxemia*”. O monitoramento precoce da saturação de oxigênio desde a fase domiciliar permitiu a antecipação do aparecimento da dispneia, que já representa um indicativo da fase adiantada do comprometimento pulmonar e da iminente necessidade de suplementação de oxigênio e/ou ventilação invasiva.^5^

A avaliação da capacidade cardiorrespiratória (ACR) ou funcional ao exercício é uma ferramenta poderosíssima na definição do estado de saúde de uma maneira geral na população, seja em indivíduos saudáveis ou com doença cardiovascular, superando outras variáveis no valor prognóstico, como a presença de isquemia miocárdica ao eletrocardiograma no esforço.^6^ Essa análise leva em conta o funcionamento integrado dos sistemas cardiovascular, respiratório, metabólico e biomecânico.

Na prática diária da cardiologia, nos consultórios, a cardiologia preventiva e a cardiologia do esporte têm ganhado bastante importância como motivo de procura da população ao médico. No arsenal de exames e avaliações, a ACR deve estar entre as principais medidas devido ao seu importante valor prognóstico. O sedentarismo representa um dos maiores males para a saúde e é um importante fator de risco para doenças cardiovasculares e mortes em geral.^7,8^ O teste de esforço, principalmente o teste cardiopulmonar (TCP) é a principal ferramenta na avaliação da ACR. A referência para a classificação de um indivíduo em relação a sua ACR deve estar de acordo com a população na qual estamos avaliando. Por muitos anos, em nosso país, utilizamos valores de referências de outras populações, de outros países, para classificarmos nossos indivíduos. Vários pesquisadores buscaram em nossas casuísticas nacionais parâmetros relativos à ACR e equações de predição de VO_2_. Em 2011, nosso grupo descreveu valores de referência em uma população de 3.992 indivíduos de ambos os sexos, ativos e sedentários, e, a partir desta população, apresentamos pela primeira vez uma proposta de classificação da ACR com base em dados de 2.837 pessoas fisicamente ativas.^9,10^ Almeida et al., utilizando dados de 2.495 indivíduos, desenvolveram uma equação de predição de VO_2_ de grande utilidade para os laboratórios de TCP nacionais.^11^

Nessa robusta compilação de Rossi Neto et al., em que 18.186 indivíduos realizam TCP em um período de 16 anos, nova proposta nacional de ACR foi apresentada.^12^Análise da distribuição dos valores de VO_2_ ao longo das faixas etárias em ambos os sexos mostra uma redução gradativa com o progredir dos anos. A idade entre 30 e 49 anos teve a maioria dos participantes mostrando a realidade da população que busca as clínicas para realização de *check-up* de saúde. A proposta apresentada usa a média populacional da amostra para elaboração dos diferentes níveis de ACR em uma população ampla de menores de 19 até 79 anos. Percebemos uma distribuição muito semelhante dos níveis *muito ruim, ruim, regular* e *bom*. Em menor número, em torno de 10%, temos os mais bem condicionados. Nota-se que nem mesmo a classificação excelente ou superior está acima da média, e também não temos informações quanto ao número de sedentários ou ativos da amostra. O presente estudo também tentou fazer comparações com outras séries, como Cooper e o estudo FRIEND, mas as diferenças na população e a dificuldade de acesso aos dados amostrais desses estudos dificultam uma completa comparação. Os valores de VO_2_ máximo de Cooper não são adequados para a comparação, por serem obtidos de forma indireta.

Uma das grandes utilidades das classificações da ACR é situar o indivíduo testado quanto ao seu estado de aptidão física frente às pessoas de sua faixa etária. Em uma comparação, podemos situar o estado funcional (idade biológica) diante de um valor de ACR relativo a uma determinada faixa etária (idade cronológica). Essa informação pode ser uma valiosíssima ferramenta no aconselhamento para a prática de exercícios e combate ao sedentarismo.

Por ser uma grande amostra de uma cidade muito populosa e muito miscigenada, o presente estudo oferece mais uma possibilidade de classificação às propostas já existentes de ACR.^10^ Em conjunto com os estudos anteriores de referência em TCP, o presente estudo coloca nosso país entre os líderes na execução do TCP, em que dados nacionais robustos e bastante representativos de diversas regiões podem ser utilizados para correta análise e classificação dessa importante informação biológica, que é a aptidão cardiorrespiratória.
