# Supplementary material for: Consumo de Oxigênio e Aptidão Cardiorrespiratória | Diferença entre Idade Cronológica e Biológica
Source: Arq Bras Cardiol. 2020 Sep 18;115(3):478–9. [Article in Portuguese] doi: 10.36660/abc.20200648 (PMC9363092; doi:10.36660/abc.20200648)
Supplement: Translation

# Translation: Oxygen Consumption and Cardiorespiratory Fitness. The Difference between Chronological and Biological Age

**Authors:** Artur Haddad Herdy, Amberson Vieira Assis

**Affiliations:** 1 Brazil Instituto de Cardiologia de Santa Catarina – Cardiologia, São José, SC – Brazil

**Keywords:** Motor Activity, Exercise, Social Class, Endurance Training, Physical Endurance, Healthy Lifestyle, Sedentarism, Myocardial Ischemia/prevention and control

Oxygen is undoubtedly one of the main elements for the life of an aerobic organism. Animals with more complex structures, including humans, developed oxidative metabolism in the evolutionary process as the main source of energy production. Therefore, our ability to consume and use oxygen represents our main power plant and is closely associated with our vitality. Its measurement should undoubtedly be considered as another vital sign. When assessing athletes or people with severe pathologies, oxygen consumption (VO_2_) is an important marker of performance or survival^1^. VO_2_ also defines essential therapies in critically-ill patients with CHF. In a series of 715 patients referred for heart transplantation, the event-free survival, such as death and transplantation in one year, was 87% with VO_2_ > 14, 77% between 10.1 and 14, and 65% when VO_2_ ≤ 10 mL/kg.min.^2,3^ On the other hand, values above 20 mL/kg.min are considered to have a better prognosis and may continue with the pharmacological therapy.^4^

Recently, in the current coronavirus-19 pandemic (Sars-CoV-2), the value of arterial oxygen levels showed to be an important tool in the assessment of the severity of these patients. In the initial cases of lung injury, patients were relatively well, despite oxygen desaturation, which is not very responsive to oxygen therapy, in a clear gap between the severity of hypoxemia and symptoms, which has been described as “Happy hypoxemia”. The early monitoring of oxygen saturation since the home phase allowed the anticipation of the dyspnea onset, which already represents an indication of the advanced stage of pulmonary impairment and the imminent need for oxygen supplementation and/or invasive ventilation.^5^

The evaluation of cardiorespiratory fitness (CRF) or functional capacity to exercise is a very powerful tool in the definition of the general health status in the population, whether in healthy individuals or those with cardiovascular disease, surpassing other variables regarding the prognostic value, such as the presence of myocardial ischemia at the electrocardiogram during exercise.^6^ It takes into account the integrated function of the cardiovascular, respiratory, metabolic and biomechanical systems.

In the daily practice of cardiology, in medical offices, preventive cardiology and sports cardiology have gained considerable importance, as a reason for the population to seek medical attention. In the arsenal of exams and evaluations, CRF should be among the main measures, due to its important prognostic value. A sedentary lifestyle represents one of the greatest health problems and is an important risk factor for cardiovascular diseases and death in general.^7,8^ The exercise test, mainly the cardiopulmonary test (CPT) is the main tool for the evaluation of CRF. The reference for the classification of an individual in relation to his CRF must be in accordance with the population in which we are evaluating. For many years, in our country, we have used reference values obtained from other populations, from other countries, to classify our individuals. Several researchers searched our national series for parameters related to CRF and VO_2_ prediction equations. In 2011, our group described reference values in a population of 3,992 individuals of both genders, active and sedentary ones, and based on that population we presented for the first time a proposal for the classification of CRF based on data from 2,837 physically active individuals.^9,10^ Almeida et al, using data from 2,495 individuals, developed a VO_2_ prediction equation that is very useful for national CPT laboratories.^11^

In this robust compilation by Rossi Neto et al, where 18,186 individuals performed CPT over a 16-year period, a new national proposal for CRF was presented.^12^ The analysis of the distribution of VO_2_ values over the age groups in both genders shows a gradual reduction with aging. The age group between 30 and 49 years had the vast majority of participants, showing the reality of the population that seeks clinics to undergo health check-ups. The presented proposal uses the population average of the sample to create the different levels of CRF in a large population sample aged from 19 to 79 years old. We noticed a remarkably similar distribution of the very bad, bad, regular and good levels. At a smaller number, around 10% have the best fitness. It was observed that the neither excellent nor higher classification is above average, and we also do not have information on the number of sedentary or active individuals in the sample. The present study also tried to make comparisons with other series, such as Cooper and the FRIEND study, but the differences in the population and the difficulty in gaining access to the sample data of these studies makes a complete comparison difficult. Cooper’s maximum VO_2_ values are not suitable for comparison because they are obtained indirectly.

One of the great uses of the CRF classifications is to situate the tested individuals in terms of their physical fitness status in relation to people from their age group. In a comparison, we can situate the functional status (biological age) against a CRF value related to a given age group (chronological age). This information can be a very valuable tool when counseling about the practice of exercises and fighting a sedentary lifestyle.

As this is a large sample from a very densely inhabited and very mixed-race city, the present study offers yet another possibility to classify the existing CRF proposals.^10^ Together with the previous reference studies in CPT, the present study places our country among the leaders in the performance of CPT, where robust national data and very representative of different regions can be used for the correct analysis and classification of this important biological information, which is cardiorespiratory fitness.
